# PCSeg: Color model driven probabilistic multiphase level set based tool for plasma cell segmentation in multiple myeloma

**DOI:** 10.1371/journal.pone.0207908

**Published:** 2018-12-12

**Authors:** Anubha Gupta, Pramit Mallick, Ojaswa Sharma, Ritu Gupta, Rahul Duggal

**Affiliations:** 1 SBILab, Department of ECE, Indraprastha Institute of Information Technology-Delhi (IIIT-Delhi), New Delhi, India; 2 Department of Computer Science, Courant Institute of Mathematical Sciences, New York University, New York City, New York, United States of America; 3 Department of CSE, Indraprastha Institute of Information Technology-Delhi (IIIT-Delhi), New Delhi, India; 4 Laboratory Oncology Unit, Dr. B. R.A. IRCH, All India Institute of Medical Sciences (AIIMS), New Delhi, India; Beijing University of Technology, CHINA

## Abstract

Plasma cell segmentation is the first stage of a computer assisted automated diagnostic tool for multiple myeloma (MM). Owing to large variability in biological cell types, a method for one cell type cannot be applied directly on the other cell types. In this paper, we present PCSeg Tool for plasma cell segmentation from microscopic medical images. These images were captured from bone marrow aspirate slides of patients with MM. PCSeg has a robust pipeline consisting of a pre-processing step, the proposed modified multiphase level set method followed by post-processing steps including the watershed and circular Hough transform to segment clusters of cells of interest and to remove unwanted cells. Our modified level set method utilizes prior information about the probability densities of regions of interest (ROIs) in the color spaces and provides a solution to the minimal-partition problem to segment ROIs in one of the level sets of a two-phase level set formulation. PCSeg tool is tested on a number of microscopic images and provides good segmentation results on single cells as well as efficient segmentation of plasma cell clusters.

## Introduction

Cell classification via image processing has recently gained interest from the point of view of building computer assisted diagnostic tools for hematological malignancies. The computer assisted image processing tools can evaluate morphological features that are not discernable with human eyes. If automated, these tools can be used to analyze large number of cells in an objective manner for reliable assessment of specific cell populations of interest. The process of ‘Cell Segmentation’ is a precursor to cell classification implemented via image processing and hence, is the first stage of any computer assisted diagnostic tool. Several methods for cell segmentation have been described in the literature and often multiple methods are combined to achieve reasonable results depending on the type of cell images. Broad categories of segmentation methods include intensity thresholding methods, region-based segmentation methods, machine learning based methods and active contour methods [1].

Intensity thresholding based segmentation is one of the simplest and fastest methods of image segmentation. Dorini et al. [2] used intensity thresholding to segment nuclei of mature lymphocytes. Sharif et al. [3] utilized information contained in YCBr color space along with intensity thresholding, morphological operations, and watershed segmentation to segment red blood cells from the microscopic images. The method of Dorrini et al. [2] fails to delineate the regions of interest (ROI) and the method of Sharif et al. [3] does not accommodate spatial intensity variation in images as it depends on the structuring element chosen. Hence, both the methods do not yield robust results, especially, when cells are present in clusters.

Region-based segmentation approaches look for connected components on the basis of properties such as texture and brightness. These approaches include seed based region growing and merging approaches [4–6]. In general, region growing methods are computationally expensive, are sensitive to noise, require correct identification of seeds, are local in nature without any global view, and at times have problem with the stopping criterion.

Machine learning based methods carry out segmentation via grouping of similar pixels (e.g. based on Euclidean distance on intensity) into clusters or by using other methods that learn pixel characteristics. Watershed, *k*-means clustering, and Support Vector Machines (SVM) are some of the most often used algorithms in segmentation [3, 7–10]. However, none of the above methods are able to segment cells of interest from cell clusters.

Active contour approach works on deformable curves that change their shapes according to the boundaries of targeted objects in an image using internal and external forces defining the motion of closed 2D contours [11–17]. Sadeghian et al. [12] carried out edge detection using Canny filter followed by geodesic snake contour method to segment leukocytes. However, these methods work on single cell windows extracted from the full microscopic image. Also, edge detection fails whenever intensity of nucleus and cytoplasm are similar.

**Fig 1:**
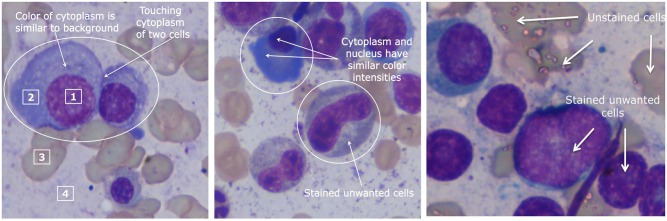
Challenges associated with plasma cell segmentation; numbers in boxes indicate the following image regions: 1 nucleus of plasma cells, 2 cytoplasm of plasma cells, 3 unstained cells, and 4 background. Three challenges are highlighted via this Fig: 1) At times, the color difference between the cytoplasm with the adjacent background is less; 2) Plasma cells may be clustered together and hence, segmentation of the overlapping/touching cells is required; and 3) there may be more than one type of stained and unstained cells posing difficulty in extracting plasma cells of interest.

With the focus of our study on building a robust automated pipeline for residual disease estimation in Multiple Myeloma (MM), a type of plasma cell (PC) cancer, the segmentation of the plasma cells was initiated as the first step. The pre-determined challenges specific to plasma cell segmentation are as follows (Fig 1): 1) Plasma cell segmentation requires segmentation of both nucleus and cytoplasm. At times, the color contrast of nucleus and cytoplasm and, more often, of the cytoplasm with the adjacent background is less due to overstaining or understaining. This poses difficulty in cell segmentation; 2) Plasma cells may be clustered together and hence, segmentation of the overlapping/touching cells is required. Generally, this is difficult because of different configurations as a) nuclei of different cells are touching, b) nuclei of one and cytoplasm of another cell are touching, or c) the cytoplasm of different cells are touching; and 3) Presence of more than one type of stained and unstained cells poses another challenge in extracting plasma cells of interest.

Although region growing and machine learning based methods have largely been used in cell segmentation, these methods are not effective in cluster segmentation [4–6, 8]. Contour based approaches such as snake models, level set models, and their variants are increasingly being used for segmentation in medical microscopic images [12–15, 17]. For example, Yang et al. [13] incorporated a color based gradient in the standard Gradient Vector Flow (GVF) model, a contour based approach to exploit the crucial information present in different histological components such as nucleus and cytoplasm of lymphocytes, follicle and mantle cells. Zamani and Safabakhsh [14] worked on a similar approach using GVF based on color gradients with the gradient flow initialized with the nuclei contours to identify nuclei using adaptive histogram thresholding to perform segmentation of lymphocytes. However, the accuracy of segmentation depends on the preliminary step of locating nuclei using histogram thresholding that is generally not robust. Also, this approach fails to segment cell clusters. Yu et al. [15] used level set by Chan-Vese [16] to first segment only the nuclei of nerve cells and later used another level set to segment complete cells (nucleus and cytoplasm). Recently, Lu et al. [17] proposed a joint level set initialized with cell nuclei for pap smear cell segmentation. However, this approach fails in regions of low contrast between the nucleus and the cytoplasm.

From the above literature review, it appears that a contour based method may be able to provide a clear boundary of cells compared to morphological, thresholding, or clustering techniques. Since most of the above contour based methods are deterministic, a probabilistic level set formulation may be able to capture intra-subject and inter-subject related intensity variations within biological components such as within the cytoplasm. Region based and machine learning based methods have largely been used in cell segmentation but these methods are not observed to be effective in cluster segmentation. Contour based approaches such as snake models or level set models are the state-of-the-art medical image segmentation methods that are increasingly being used for segmentation in medical microscopic images [12–15, 17–19] as well as in other medical imaging applications, say, CT segmentation and brain MRI segmentation [20–23].

This motivates us to explore level set formulation within the probabilistic framework for plasma cell segmentation including cluster segmentation from microscopic images. In the present study, the existing methods as well as a recently described method by Saeedizadehet al. [24], using combination of thresholding, modified bottleneck algorithm, and watershed to segment plasma cells, was evaluated for segmentation of plasma cells in our set-up. The ultimate purpose of our work is to build an automated multiple myeloma residual disease detection tool for deployment in the hospital. Incorrect segmentation or partial segmentation of PCs will hinder the development of the subsequent classifier. Thus, we were motivated to explore the problem of PC segmentation afresh for robust results.

## Materials and methods

Microscopic images were captured from bone marrow aspirate slides of patients diagnosed with multiple myeloma as per the standard guidelines [25]. Slides were stained using Jenner-Giemsa stain. Images were captured at 1000x magnification using Nikon Eclipse-200 microscope equipped with a digital camera. Images were captured in raw BMP format with a size of 2560x1920 pixels. In all, our dataset consisted of 85 images. We trained our pipeline on 15 images. All the images were stain normalized, using the methodology proposed earlier [26], before being used for segmentation.

Written informed consent was obtained from all the subjects as per the guidelines of the Institute Ethics Committee (IEC) of All India Institute of Medical Sciences (AIIMS), New Delhi, India (Approval No. IEC/NP-145/2013 & RP-32/06.05.2013). Subsequently, a waiver for written informed consent for obtaining photomicrographs from the bone marrow aspirate slides was taken from IEC (approval No. OP-06/01.12.2017). One of the co-authors (RG) had access to the patient identifying information which was completely removed from the image data sets before sharing of data with the other co-authors for building up the PCSeg tool presented in this paper. The dataset is available at the public repository [37].

For the purpose of segmentation, an MM image can be divided into four regions of interest (ROI): (1) nucleus of PC, (2) cytoplasm of PC, (3) unstained cells, and (4) background (Fig 1). For efficient segmentation of all the four ROI, PCSeg Tool has been designed with the following four steps:

Step-1: Statistical modeling and computation of separability index of the four ROI in the images
Step-2: Removal of unstained cells
Step-3: Extraction of nucleus and cytoplasm of plasma cells using the proposed multiphase level set methodology
Step-4: Cluster cell segmentation using watershed and circular Hough transform

### Step-1: Statistical modeling and computation of separability index of regions of interest

First, the statistical characterization of the four ROI of MM images, i.e., nucleus of PC, cytoplasm of PC, unstained cells, and background (Fig 1) was done and the intensity profile of these regions was studied in different color spaces. A set of fifteen reference MM images, representative of the color histograms, were chosen and the histograms of RGB, HSV, and Lab color channels of the four ROI were marked in these reference images as shown in Fig 1. Since images were at a very high resolution of 2560 x 1920, sufficient numbers of pixels were available for computing the histogram. Histograms of RGB, HSV, and Lab color channels of the four ROI and their corresponding Gaussian probability density functions (PDFs) were fitted to the normalized histograms.

**Fig 2:**
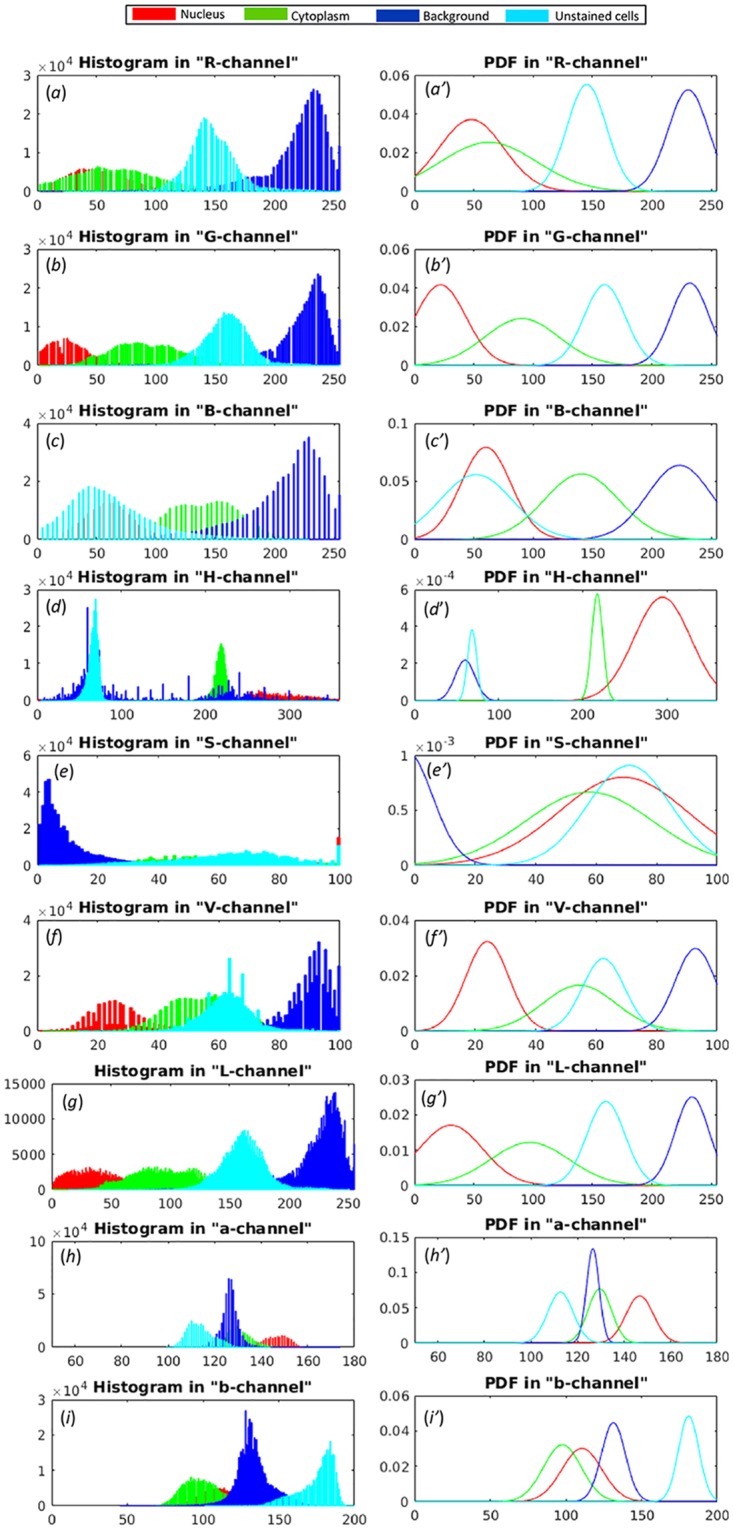
**Histogram of four image regions (nucleus, cytoplasm, background, and unstained cells) in:**(a)-(c) RGB, (d)-(f) HSV, and (g)-(i): Lab color spaces; Corresponding fitted probability density functions (PDFs): (a′)-(c′) RGB, (d′)-(f′) HSV, and (g′)-(i′): Lab color spaces.

Fitted PDFs are drawn in Fig 2 in the intensity ranges of the original histograms. It is evident from these histograms that 1) nucleus and cytoplasm overlap in every color channel, although this overlap is considerably less in blue (B), hue (H), and value (V) channels; 2) nucleus and cytoplasm appear considerably separated from the background in red (R) and green (G) channels; and 3) the unstained cells do not overlap with nucleus and cytoplasm of plasma cells in the hue (H) channel. Thus, although it may be possible to remove unstained cells using the intensity profile in H-channel, nucleus and cytoplasm of plasma cells cannot be discerned using any single color channel. Rather, a combination of color channels would be required to separate these.

In order to quantify the separability of different image regions using probability distributions in RGB, HSV, and Lab color spaces, we used Bhattacharyya distance (*D*_*B*_) as a metric that quantifies separation between two PDFs *p* and *q* [27] as:

$$\begin{array}{c} D_{B}\left(p,q\right)=-\log_{e}(\int \sqrt{p(x)q(x)}\text{d}x). \end{array}$$

**Fig 3:**
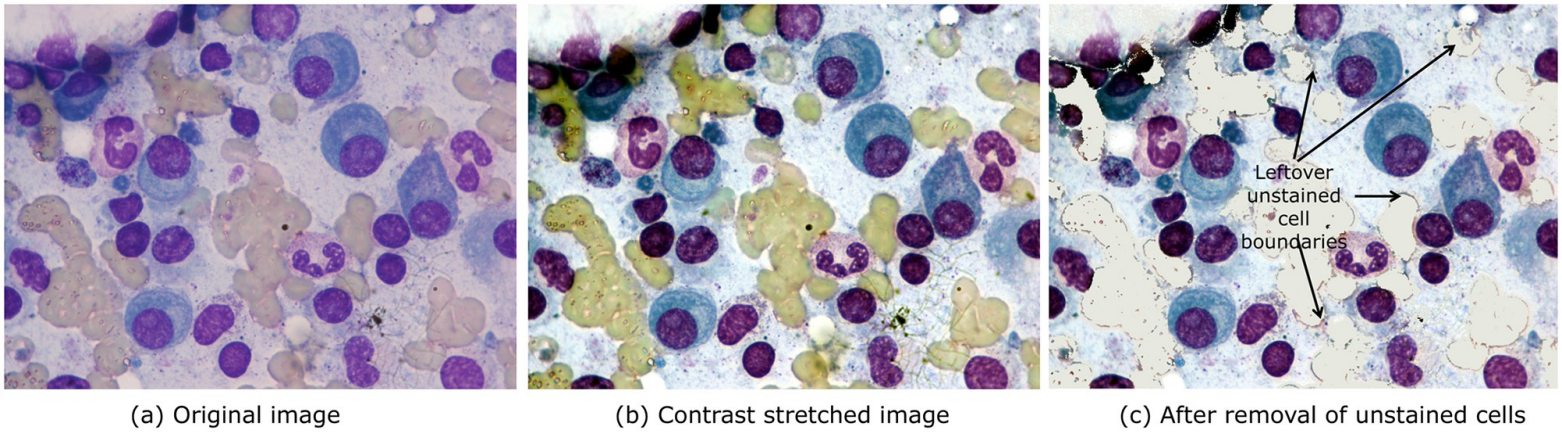
Contrast stretching followed by unstained cell removal, image patch size 2560 × 1920 this figure shows (a) an original image that is (b) contrast stretched such that 1% of lower and higher intensity values are saturated to 0 and 255, respectively. From the probability density functions of the four regions of interest (nucleus of PC, cytoplasm of PC, unstained cells, and background) of the resulting contrast stretched images, it is observed that nucleus and cytoplasm of plasma cells have maximum separability with unstained cells in H color channel. Unstained cells are removed by replacing intensity of pixels having values less than 120 in the H-channel with the background pixel intensity leading to (c).

Prior to computing *D*_*B*_, we applied contrast stretching on the RGB image such that 1% of lower and higher intensity values are saturated to 0 and 255, respectively. Next, we converted this contrast stretched image (Fig 3b) to HSV and Lab color spaces. The distance between all required combinations of two ROI in RGB, HSV, and Lab color spaces were computed (Table 1). These values are indicative of the separability between different regions and are used in the proposed modified multiphase level set method.

**Table 1:** Bhattacharyya distance calculated between different image regions using the ground truth data.

Color Channels	Bhattacharyya distance between two regions of interest					
	PC Nucleus and PC Cytoplasm	PC Nucleus and Background	PC Nucleus and Unstained Cells	PC Cytoplasm and Background Cells	PC Cytoplasm and Unstained Cells	Background and Unstained Cells
R	0.06	8.27	2.38	3.86	1.06	3.31
G	0.85	**15.89**	6.27	**4.21**	1.02	2.38
B	1.17	5.74	0.04	1.00	1.03	4.44
H	**1.76**	1.10	**11.19**	1.26	**73.13**	1.09
S	0.02	0.96	0.03	0.7	0.08	1.31
V	1.25	14.48	3.75	2.17	0.15	2.85
L	0.68	7.67	3.30	2.52	0.57	3.05
a	0.87	2.09	4.74	0.18	1.54	1.97
b	0.14	0.45	4.38	1.28	6.70	**4.89**

### Step-2: Removal of unstained cells

It is observed that both nucleus and cytoplasm of plasma cells have maximum separability with unstained cells in H color channel with large values of *D*_*B*_ distance (Table 1). Thus, we identified unstained cells using the H-color channel. Since both background and unstained cells are unwanted regions for the purpose of plasma cell segmentation, we replaced unstained cell pixels with the background pixels. This is carried out by replacing intensity of pixels having values less than 120 in the H-channel with the background pixel intensity (Fig 2). This replacement of unstained cells’ intensity with the background intensity ensured that no additional region is created for the subsequently used multiphase level set algorithm (Fig 3(c)). Although unstained cells were removed, some outliers were still left (Fig 3(c)) that were subsequently removed in Step-3 of the proposed algorithm.

### Step-3: Stained cell extraction using the proposed modified multiphase level set method

We modified the multiphase level set formulation by utilizing statistical information of the four ROI in the image, i.e., nucleus, cytoplasm, unstained cells, and the background, wherein the four ROI were modeled via four-phases of two level sets. Each of the ROI was assigned one phase and the corresponding label. We assigned labels Ω_11_ and Ω_10_ to the first level set *ϕ*_1_ for the nucleus and cytoplasm, respectively. Likewise, we assigned labels Ω_01_ and Ω_00_ to the second level set *ϕ*_2_ for the background and the remaining unstained cells, respectively. Next, four probability maps of the entire image were created corresponding to each of the four phases of the level set (one each for their respective regions of interest, namely, nucleus, cytoplasm, unstained cells, and the background) as below:

$$\begin{array}{c} p\left(U_{0}|\Omega_{ij}\right)=\frac{\sum_{c=1}^{9}w_{c,\Omega_{ij}}*p\left(U_{0,c}|\Omega_{ij}\right)}{\sum_{c=1}^{9}w_{c,\Omega_{ij}}}, \end{array}$$ (1)

where *U*_0_ is the contrast stretched image and *c* corresponds to color channels with *c* = 1, 2, 3, 4, …, 8, 9 for channels R, G, B, H, S, V, L, a, and b, respectively. *U*_0,*c*_ is the *c*^*th*^ color channel of the image *U*_0_, Ω_*ij*_ is one of the four ROI with *i*, *j* ∈ {0, 1}, $w_{c,\Omega_{ij}}$ are the weights, *p*(*U*_0,*c*_|Ω_*ij*_) is the conditional probability (over the ROI Ω_*ij*_) of the color channel image, and *p*(*U*_0_|Ω_*ij*_) is the conditional probability of the original image over all four phases constructed using the weighted probability in all color channels.

Bhattacharyya distance *D*_*B*_ in Table 1 was used to determine weights because it provides an appropriate metric for discerning ROI. Weights for a channel were assigned based on the ability of discerning the desired ROI from all other ROI in that channel. Since Bhattacharyya distance would be higher for larger separation, it can be used as the weight, provided this distance is larger than some minimum threshold. For example, nucleus is discernible from both cytoplasm and background in blue channel with *D*_*B*_ > 1 for each ROI (Table 1). Hence, the maximum of the two distances (distance between nucleus and cytoplasm, and distance between nucleus and background) is chosen as the weight for nucleus in blue channel. On the other hand, nucleus cannot be separated from cytoplasm in the red channel, although it is widely separated from the background in this color channel. This implies that nucleus cannot be extracted from all other ROI in red channel and hence, a zero weight is chosen for nucleus in this channel. Likewise, weights were chosen in stepwise manner for all the ROI, as detailed below.

For determining weights, $w_{c,\Omega_{11}}$, in (1) for nucleus, a channel was chosen (R, G, B, H, S, V, L, a, or b) and if the Bhattacharya distance *D*_*B*_ (Table 1) between both 1) nucleus and cytoplasm, and 2) nucleus and background was greater than 1, the maximum distance of the above two was chosen as the weight for nucleus in that channel. Else a value of zero was assigned to the weight in that channel (2). The process was repeated for all the channels.

wc,Ω11={max[DB(p(U0,c|Ω11),p(U0,c|Ω10)),DB(p(U0,c|Ω11),p(U0,c|Ω01))]ifDB(p(U0,c|Ω11),p(U0,c|Ω10))>1andDB(DB(p(U0,c|Ω11),p(U0,c|Ω01))>10otherwise (2)

Similarly, weights were assigned for cytoplasm based on the Bhattacharya distance *D*_*B*_ between both 1) cytoplasm and nucleus, and 2) cytoplasm and background in each of the channels (3).

wc,Ω10={max[DB(p(U0,c|Ω10),p(U0,c|Ω11)),DB(p(U0,c|Ω10),p(U0,c|Ω01))]ifDB(p(U0,c|Ω10),p(U0,c|Ω11))>1andDB(DB(p(U0,c|Ω10),p(U0,c|Ω01))>10otherwise (3)

Weights were assigned for background based on the Bhattacharya distance *D*_*B*_ between both 1) background and nucleus, and 2) background and cytoplasm. Weights were assigned for unstained cells based on the Bhattacharya distance *D*_*B*_ between both 1) unstained cells and nucleus, and 2) unstained cells and cytoplasm. Since plasma cells are required to be clearly delineated from the background and unstained cells, a greater threshold of 3 was considered for background (4) and unstained cells (5).

wc,Ω10={max[DB(p(U0,c|Ω01),p(U0,c|Ω11)),DB(p(U0,c|Ω01),p(U0,c|Ω10))]ifDB(p(U0,c|Ω01),p(U0,c|Ω11))>3andDB(DB(p(U0,c|Ω01),p(U0,c|Ω10))>30otherwise (4)

wc,Ω00={max[DB(p(U0,c|Ω00),p(U0,c|Ω11)),DB(p(U0,c|Ω00),p(U0,c|Ω10))]ifDB(p(U0,c|Ω00),p(U0,c|Ω11))>3andDB(DB(p(U0,c|Ω00),p(U0,c|Ω10))>30otherwise (5)

Final weights ($w_{c,\Omega_{ij}}$) obtained using the above scheme are summarized in Table 2.

**Table 2:** Weights of each ROI for level set equations.

Color channels (*c*)	Weights ($w_{c,\Omega_{ij}}$)			
	Region Ω_11_ (Nucleus)	Region Ω_10_ (Cytoplasm)	Region Ω_01_ (Background)	Region Ω_00_ (Unstained cells)
R	0	0	8.27	0
G	0	0	15.89	0
B	5.74	1.17	0	0
H	1.76	1.76	0	73.13
S	0	0	0	0
V	14.48	2.17	0	0
L	0	0	0	0
a	0	0	0	0
b	0	0	0	6.70

Next, we defined an energy functional *E*_*p*_(*ϕ*_1_, *ϕ*_2_) for the multiphase level set formulation, where *ϕ*_1_ and *ϕ*_2_ are the two level set functions that capture the curves of cell boundaries. The energy functional *E*_*p*_(*ϕ*_1_, *ϕ*_2_) utilizes the above constructed probability maps and was added to the overall functional required to be minimized for the derivation of level set equations.

$$\begin{array}{c} \begin{array}{cc} E_{p}\left(\phi_{1},\phi_{2}\right)= & -\int_{\Omega }\log_{e}\left(p\left(U_{0}\left(x\right)|\Omega_{11}\right)\right)H(\phi_{1}\left(x\right))H(\phi_{2}\left(x\right))\text{d}x\\ & -\int_{\Omega }\log_{e}\left(p\left(U_{0}\left(x\right)|\Omega_{10}\right)\right)H(\phi_{1}\left(x\right))\left[1-H(\phi_{2}\left(x\right))\right]\text{d}x\\ & -\int_{\Omega }log_{e}\left(p\left(U_{0}\left(x\right)|\Omega_{01}\right)\right)\left[1-H(\phi_{1}\left(x\right))\right]H(\phi_{2}\left(x\right))\text{d}x\\ & -\int_{\Omega }\log_{e}\left(p\left(U_{0}\left(x\right)|\Omega_{00}\right)\right)\left[1-H(\phi_{1}\left(x\right))\right]\left[1-H(\phi_{2}\left(x\right))\right]\text{d}x, \end{array} \end{array}$$ (6)

where *H* is the Heaviside function, $\Omega \subset R^{2}$ is an open and bounded domain, $U_{0}:\Omega \mapsto R$ is the given bounded function representing the initial image, and *C* as the closed subset in Ω made up of finite set of smooth curves.

We also defined another energy functional, namely, distance energy functional *E*_*d*_(*ϕ*_1_, *ϕ*_2_) that measures the intensity difference of a given pixel from each of the region’s mean color value. To this end, we first defined and computed the distance images $U_{d,\Omega_{ij}}$ for each of the regions in (7) as:

$$\begin{array}{c} U_{d,\Omega_{ij}}\left(x\right)=[\frac{\sum_{c=1}^{9}w_{c,\Omega_{ij}}{\left(U_{0,c}\left(x\right)-\mu_{c,\Omega_{ij}}\right)}^{2}}{\sum_{c=1}^{9}w_{c,\Omega_{ij}}}], \end{array}$$ (7)

where $\mu_{c,\Omega_{ij}}$ is the mean color value of the distribution in region Ω_*ij*_ and $w_{c,\Omega_{ij}}$ is the weight of color channel *c* in region Ω_*ij*_ as tabulated in Table 2. Accordingly, we defined the distance energy functional *E*_*d*_(*ϕ*_1_, *ϕ*_2_) in (8) as:

$$\begin{array}{c} \begin{array}{cc} E_{p}\left(\phi_{1},\phi_{2}\right)= & \int_{\Omega }U_{d,\Omega_{11}}\left(x\right)H(\phi_{1}\left(x\right))H(\phi_{2}\left(x\right))\text{d}x\\ & +\int_{\Omega }U_{d,\Omega_{10}}\left(x\right)H(\phi_{1}\left(x\right))\left[1-H(\phi_{2}\left(x\right))\right]\text{d}x\\ & +\int_{\Omega }U_{d,\Omega_{01}}\left(x\right)\left[1-H(\phi_{1}\left(x\right))\right]H(\phi_{2}\left(x\right))\text{d}x\\ & +\int_{\Omega }U_{d,\Omega_{00}}\left(x\right)\left[1-H(\phi_{1}\left(x\right))\right]\left[1-H(\phi_{2}\left(x\right))\right]\text{d}x. \end{array} \end{array}$$ (8)

Adding the regularization terms of length and area, the proposed modified multiphase level set energy functional is defined in (9) as:

$$\begin{array}{c} \begin{array}{cc} E(\phi_{1},\phi_{2})= & \eta_{1}E_{p}\left(\phi_{1},\phi_{2}\right)+\eta_{2}E_{d}\left(\phi_{1},\phi_{2}\right)+\alpha_{1}\int_{\Omega }H(\phi_{1}\left(x\right))\text{d}x\\ & +\alpha_{2}\int_{\Omega }H(\phi_{2}\left(x\right))\text{d}x+\beta_{1}\int_{\Omega }|\nabla H(\phi_{1}\left(x\right))|\text{d}x\\ & +\beta_{2}\int_{\Omega }|\nabla H(\phi_{2}\left(x\right))|\text{d}x, \end{array} \end{array}$$ (9)

where *α* is a constant regularizer that controls the area inside the contour *C*, *β* controls the length of the contour and, *η*_1_ and *η*_2_ are the constants that control relative weighting of the two energy functionals. The level set (*ϕ*_1_, *ϕ*_2_) is periodically re-initialized to the signed distance function [28].

**Fig 4:**
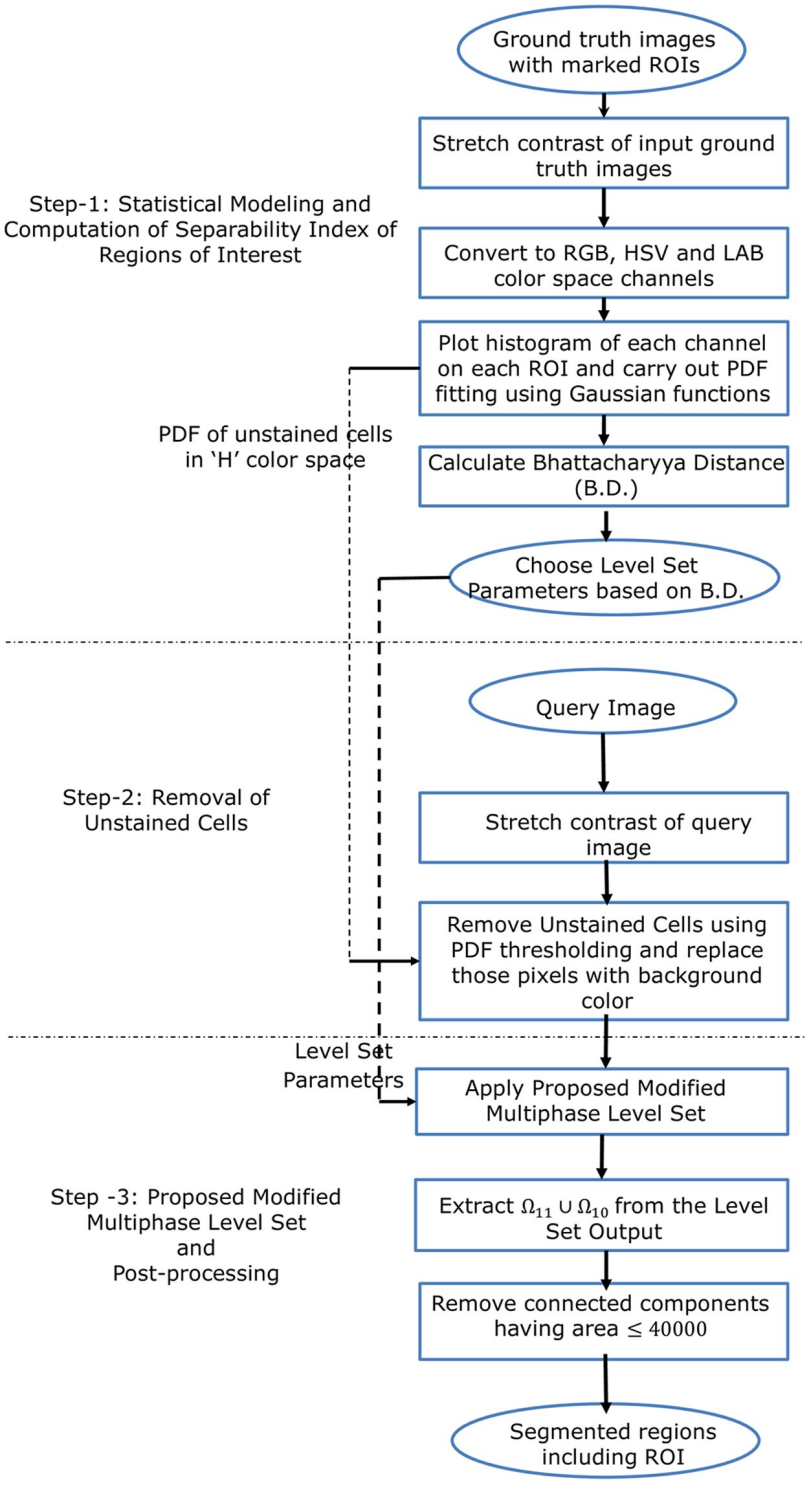
**Schematic diagram of Steps 1 to 3 of the proposed method of PCSeg tool:** Regions of interest (ROI) are: Nuclei of Plasma Cells (Ω_11_), Cytoplasm of Plasma Cells (Ω_10_), unstained cells (Ω_01_), and background (Ω_00_).

**Fig 5:**
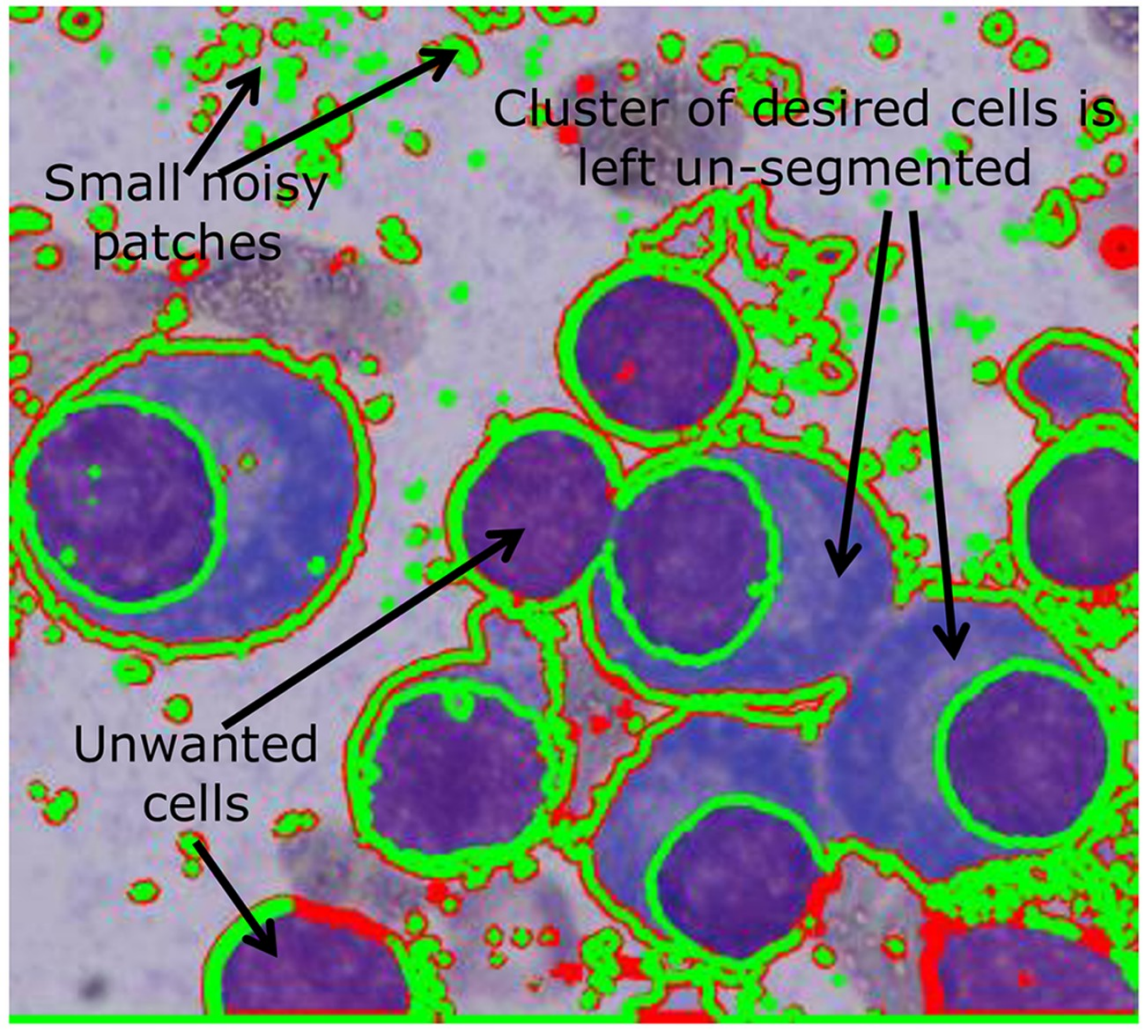
Output of the modified multiphase level set method after Step-3 on an image patch. It was noted that some unwanted stained cells (e.g. lymphocytes) were segmented to final output. In addition, some small disconnected components that were noisy patches owing to faulty manual staining were also captured by the multiphase level set. Also plasma cell clusters were not segmented.

Fig 4 presents Steps 1 to 3 of the proposed method including the modified multiphase level set formulation. The extracted mask of Ω_11_ ∪ Ω_10_ from the level set output provides the segmented plasma cells. Fig 5 presents the segmentation results after processing the input image with Steps-1 to 3.

On evaluation of the output of the multiphase level set step in Fig 5, it was noted that some unwanted stained cells, such as lymphocytes, were segmented to final output. On careful observation, we noted that the cytoplasm of stained PCs covered large cell area compared to unwanted stained cells and therefore, unwanted cells could be rejected using a threshold on cytoplasm cell area. In addition, some small disconnected components that were noisy patches owing to faulty manual staining were also captured by the multiphase level set. These small noisy disconnected components that are too small to form any ROI were rejected at the output of level set by thresholding on the size of the component.

Although all the four ROI were captured by the multiphase level set, plasma cell clusters were not segmented as observed from Fig 5. To address this problem, Step-4 was added to the tool as detailed below.

### Step-4: Cluster cell segmentation using watershed and circle Hough transform

Since stained plasma cells are approximately circular in shape, a combination of watershed and circular Hough transform (CHT) was applied to segment PC clusters. First, it is necessary to segment nuclei as the cases of touching nuclei will lead to improper cell segmentation. The nucleus of a plasma cell has a few distinct features as: 1) the nucleus is dark colored, 2) it is differently colored than the background, and 3) it is always encapsulated within the cytoplasm. Thus, the center of the nucleus could serve as an ideal seed for the watershed algorithm and the nuclei mask obtained as an output of Ω_11_ phase of the level set could be used to compute the distance transform required by the watershed algorithm.

**Fig 6:**
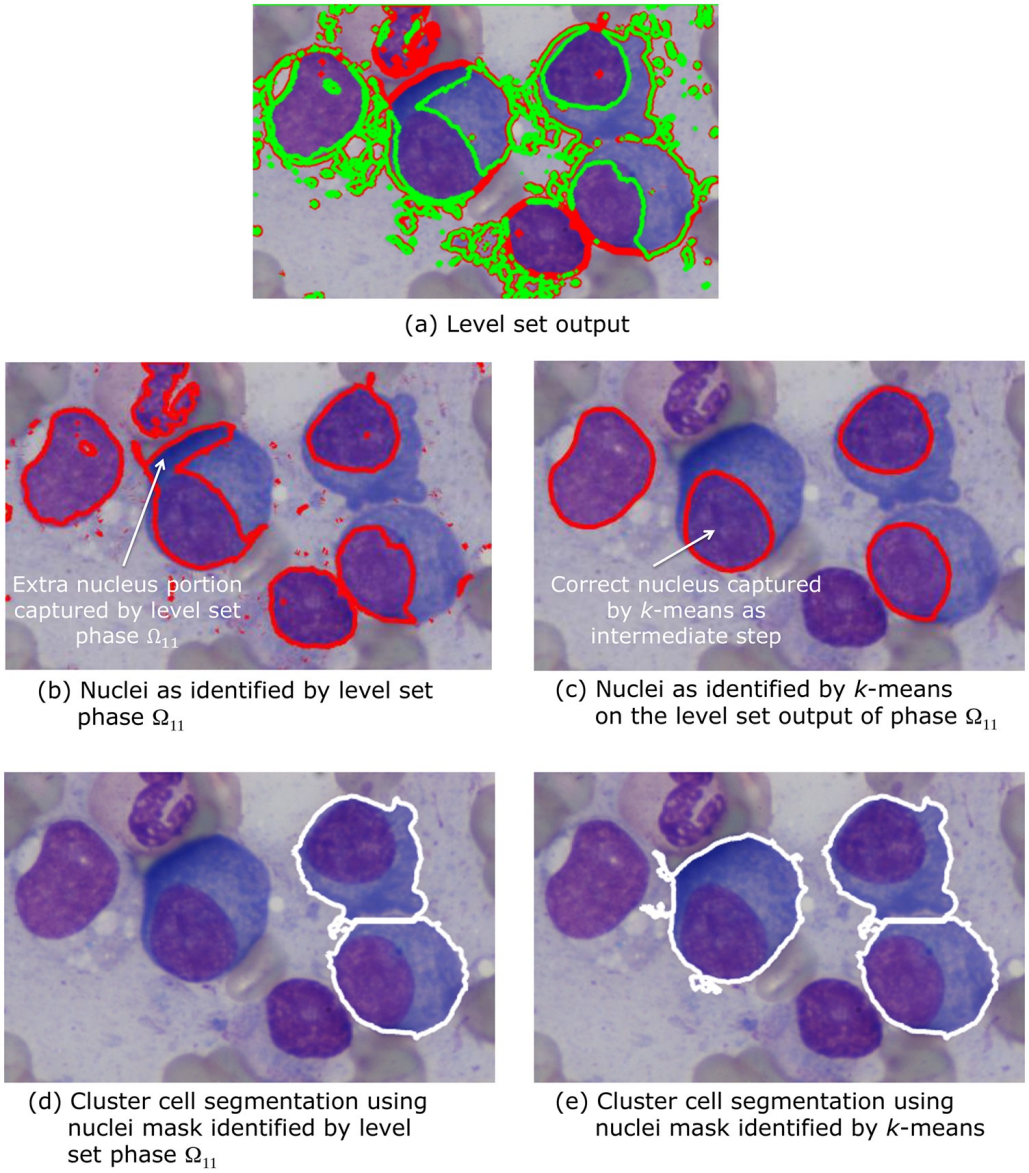
Output of cluster cell segmentation on an image patch Subfigure (a) shows the level set output wherein red boundary shows nucleus of PC being captured by levelset and green boundary shows cytoplasm of PC being captured. However, the cluster of cells are not segmented. (b) shows nuclei identified by levelset phase Ω_11_ and the problem therein of some extra mask of cytoplasm in nucleus. (d) shows cluster cell segmentation using nuclei mask identified by level set Ω_11_ in (b). One cell is falsely rejected. (c) shows nuclei identified by *k*-means on the levelset phase Ω_11_ and (e) shows correct cluster cell segmentation using the mask of (c).

However, due to the variability in cytoplasm staining, the Ω_11_ phase was observed to capture nuclei regions more liberally in some images as shown in Fig 6b. This led to unnecessary rejections of some PCs as shown in Fig 6d. Since it is vital for any medical imaging work to segment as many correct cells as possible, *k*-means (instead of Ω_11_ phase of the level set) was used to extract the nuclei mask (Fig 6c) and distance transform was applied on this mask. This basin was used by the watershed algorithm to segment the nuclei from clusters. From the watershed output, only those segmented nuclei regions were retained that were circular in nature, i.e., segmented regions that contained a center point of CHT. The non-circular regions identified as nuclei were discarded.

Following this, the distance transform of the mask of the stained portions (Ω_11_ ∪ Ω_10_) was obtained. The centers of the segmented nuclei obtained from *k*-means above were used to impose a minima on this basin and subsequently used by the watershed algorithm to segment full plasma cells from clusters. Again, we retained only those segmented regions as cells that were circular in nature, i.e., segmented regions that contained a center point of CHT.

**Fig 7:**
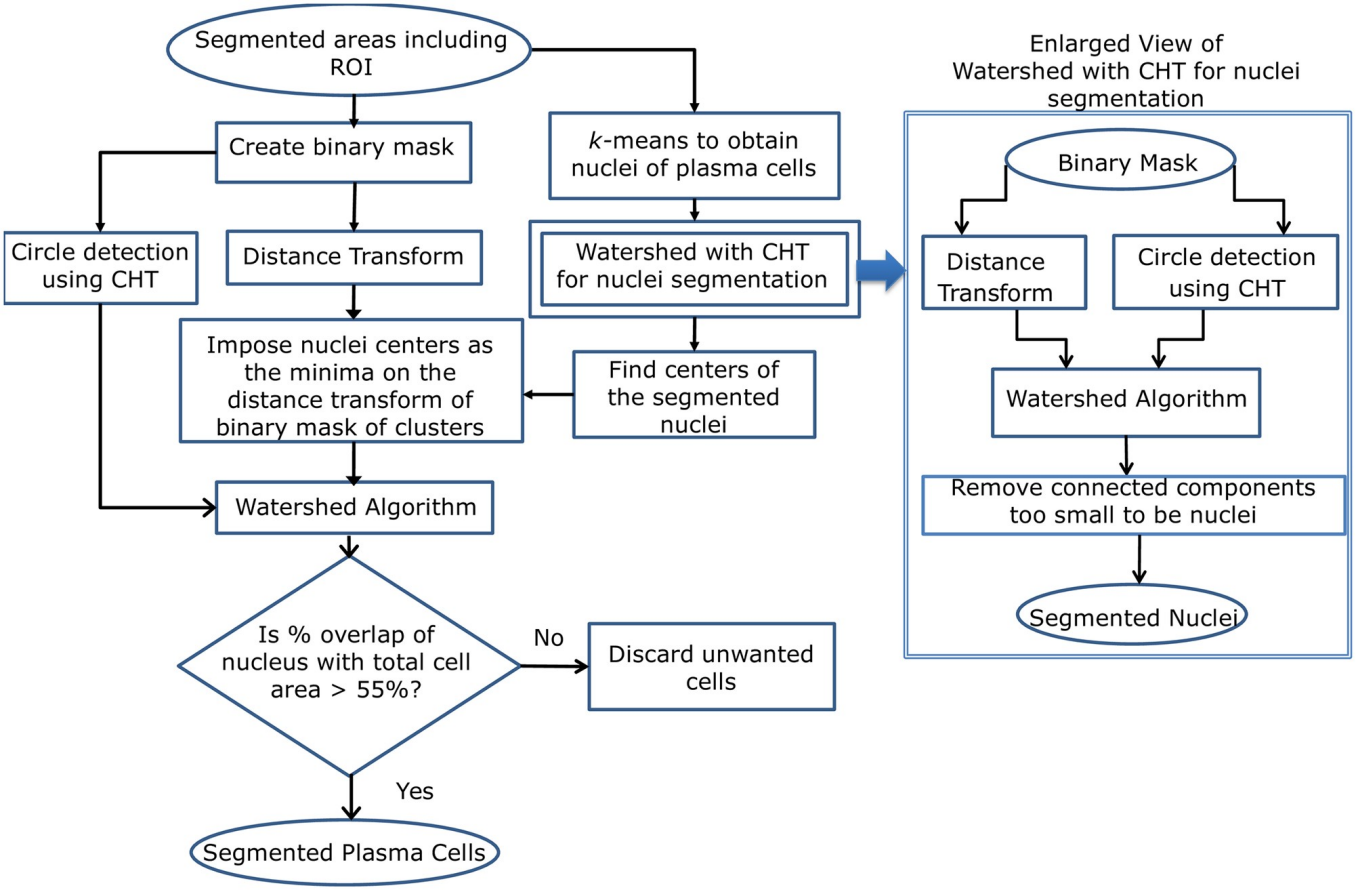
Schematic diagram of cluster cell segmentation (Step 4 of PCSeg tool) using watershed and circle Hough transform (CHT).

We have named the developed tool as PCSeg Tool-1 for the complete pipeline with *k*-means based nuclei mask for cluster cell segmentation in Step-4 and named the developed tool as PCSeg Tool-2 for the complete pipeline with Ω_11_ phase based nuclei mask for cluster cell segmentation in Step-4. The complete process pipeline of cluster cell segmentation with PCSeg Tool-1 is shown in Fig 7.

We noted that while most PCs were segmented, some of the stained lymphocytes and unwanted regions were also retained. In general, the amount of cytoplasm in lymphocytes is considerably less in comparison to PCs. Thus, for each segmented region, the ratio of the nucleus area to the total cell area was used to detect and discard these unwanted cells. As stated earlier, it was observed that the use of nuclei segmented from the phase Ω_11_ led to inadvertent rejection of some PCs (Fig 6d), while the use of nuclei segmented from *k*-means helped us in retaining such cells of interest (Fig 6e). Hence, PCSeg Tool-1 based segmentation pipeline appeared more useful. Fig 6e shows the correctly segmented cluster, while the complete process pipeline is shown in Fig 7.

### Experimental set-up

**Fig 8:**
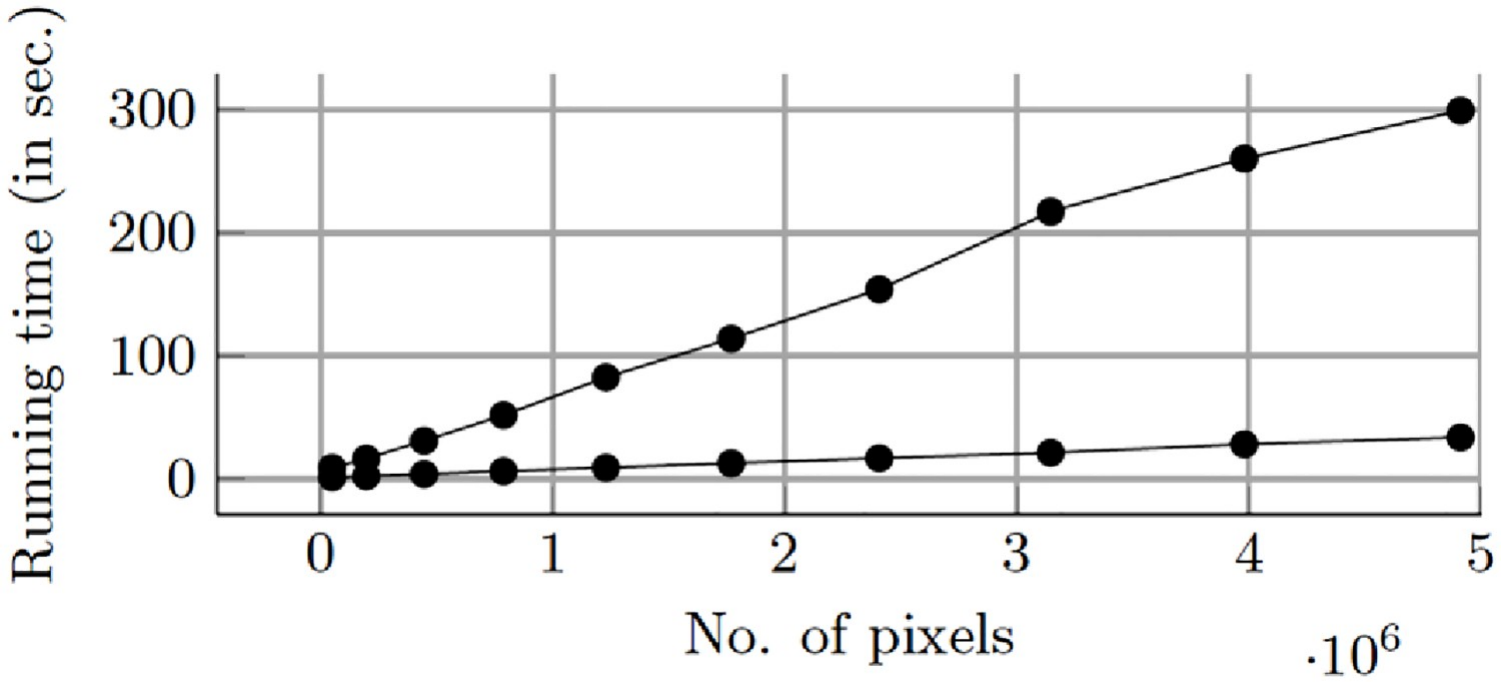
Running times on CPU and GPU.

All experiments were performed on a Ubuntu 14.04 system with an Intel^®^ Xeon(R) CPU E5-2630 v2 @ 2.60GHz 12 processor and a GeForce GTX 980/PCIe/SSE2 graphics card supporting CUDA. Level set results depend on the initialization of *ϕ*’s and therefore, initial contour was set to small circles covering the entire image to ensure faster convergence. The initialization and the energy functionals were calculated on the CPU. The level set propagation and the re-initialization of *ϕ*’s was implemented on GPU using MEX compiled files containing CUDA code. While the implementation was memory bound, the code achieved upto 75x speed up as compared to the MATLAB R2015b implementation. The rest of the pipeline was implemented in MATLAB. The GPU versus CPU computational speed results are shown in Fig 8.

## Evaluation metrics

A total of 85 images were considered, where parameters were fine tuned on 15 randomly chosen images. For our experiments, the level set parameters *α*_1_ = *α*_2_ = 0, *β*_1_ = 2, *β*_2_ = 1, *η*_1_ = 1 and *η*_2_ = 3 provided the best results. Parameters *α*_1_ and *α*_2_ are related to the compactness and the area of level set phase captured at the end. Since our images contained both isolated single cells as well as cluster of cells, putting constraints on the area provided poor results. Hence, we chose these parameters to be zero. Parameters *β*_1_ and *β*_2_ control the tightness of the boundary. Since Level set 1 captures the desired plasma cell, we wanted its boundary to be tighter compared to that of Level set 2. Hence, a higher *β* value was chosen for Level set 1 compared to Level set 2, i.e., *β*_1_ was chosen to be greater than *β*_2_. Since we used stain normalized images, the mean color vector based energy functional captured cells of interest neatly, while the probability based energy functional term took care of the slight color variations owing to subject variability and/or other variability. Hence, *η*_2_ ≥ *η*_1_ provided us best results.

The image dataset evaluated contained 260 single cells and 45 clusters in total. These 45 clusters had a total of 102 cells, with each cluster having two or more cells. For a quantitative assessment of the proposed pipeline and the method, we used TPR (True Positive Rate) or Recall rate defined as

$$\begin{array}{c} \text{TPR}=\frac{TP}{TP+FN}, \end{array}$$

PPV (Positive Predictive Value) or Precision defined as

$$\begin{array}{c} \text{PPV}=\frac{TP}{TP+FP}, \end{array}$$

and F_1_-Score defined as

$$\begin{array}{c} {\text{F}}_{1}-\text{Score}=\frac{2*precision*recall}{precision+recall}, \end{array}$$

where TP, TN, FP, and FN stand for true positive (PC detected and segmented as PC), true negative (non-PC rejected), false positive (non-PC segmented as PC) and false negative (PC rejected as non-PC), respectively. In TP or true positives, we only considered those plasma cells that were completely segmented from the image. Any plasma cell that was over-segmented or segmented out with partial portion was discarded. This is to note that F_1_- Score is same as the Dice coefficient that is used as a standard metric to assess the performance of segmentation.

Since the rate of detection of false positives is also crucial in medical applications, we evaluated False Discovery Rate (FDR) in our samples as

$$\begin{array}{c} \text{FDR}=\frac{FP}{TP+FP}. \end{array}$$

## Results

**Fig 9:**
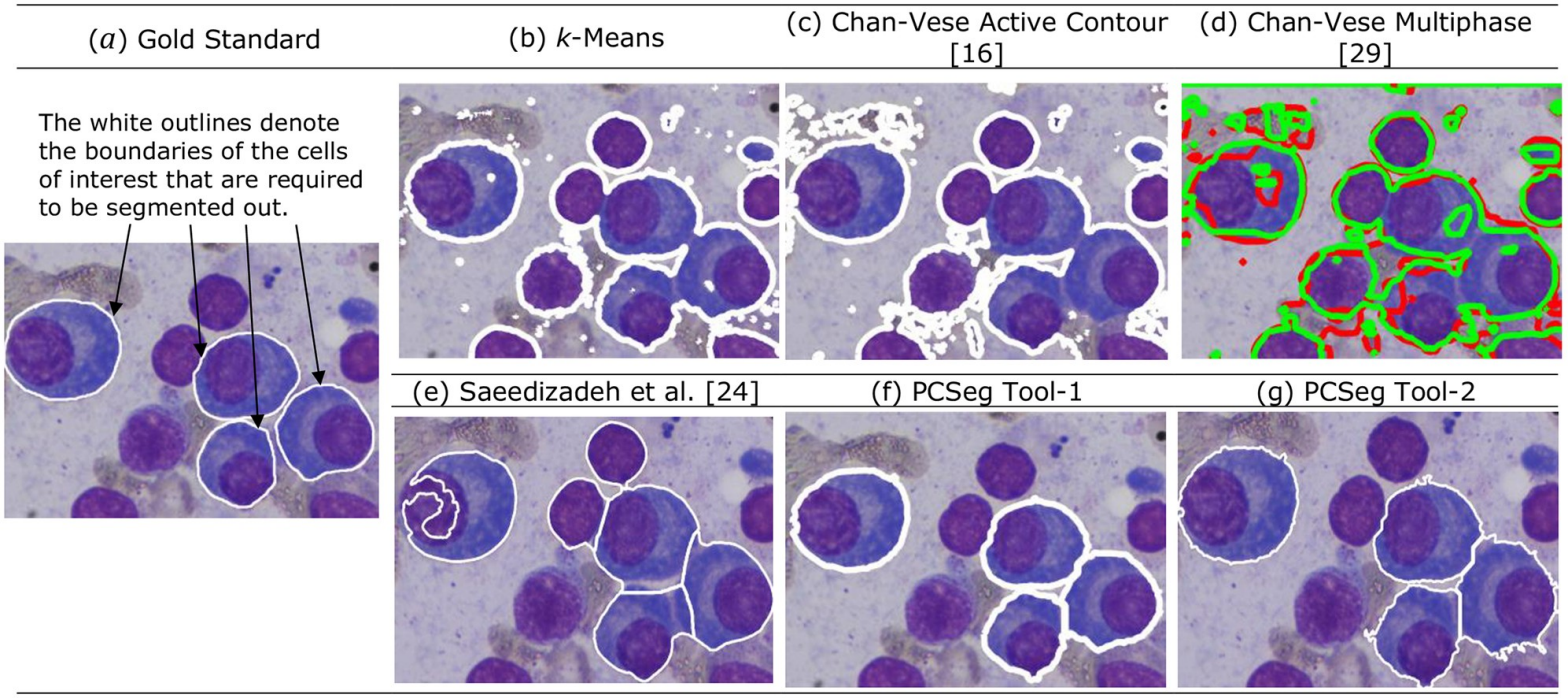
**Qualitative comparison of MM cell segmentation using different methods:** (a) Gold standard (showing cells of interest with white outlines), (b) *k*-means, (c) Chan-Vese active contour method [16], (d) Chan-Vese multiphase method [29], (e) Saeedizadeh et al. [24] method, (f) PCSeg Tool-1, and (g) PCSeg Tool-2. All white outlines in (b)-(g) denote the outlines of regions segmented out. These regions are required to be compared with the regions contained in the Gold Standard shown in (a).

We compared the results with the traditional levelset, multiphase levelset and *k*-means in Fig 9. From Fig 9, we notice that the output of these methods do not yield correctly segmented cells (including both nucleus and cytoplasm). Hence, quantification of results will not yield any accuracy with these methods that is worth comparison. Since the work by Saeedizadeh et al. [24] specifically addresses the problem of plasma cell segmentation, its pipeline is tuned to this cell type. Hence, of the existing methods including levelset, we could quantify results of this method only and thus, used the method by Saeedizadeh et al. [24] as the state-of-the-art work as of today for comparison on the given problem statement.

Quantitative results obtained on 260 numbers of isolated single PC and 45 clusters (with 102 PC) are tabulated in Table 3 and the statistical quantities on TPR, PPV, F_1_-score, and FDR are tabulated in Table 4. The PCSeg Tool-1 correctly segmented 83.5% of single and isolated plasma cells and 93.3% of PC clusters (71.1% complete clusters and 22.2% partial clusters). PCSeg Tool-2 performed slightly inferior and segmented 55.8% of single plasma cells and 64.5% of PC clusters (48.9% complete clusters and 15.6% partial clusters). Further, the number of false positives detected with PCSeg Tool-1 were about 90 cells leading to an FDR of 23.44% (Table 4). Compared to this, PCSeg Tool-2 detected 102 false positives and thus, had a higher FDR of 34.11% (Table 4). Thus, the PCSeg Tool-1 performed better than PCSeg Tool-2 in terms of both TPR and FDR.

**Table 3:** Quantitative results on plasma cell segmentation.

Method	Performance on 260 single/isolated cells	Performance on clusters, Out of (45 clusters, 102 cells in 45 clusters)		*completely rejected clusters	False positives
	Correctly segmented cells	(completely segmented clusters, completely segmented cells from clusters)	(partially segmented clusters, completely segmented cells from these clusters)		
PCSeg Tool-1	**217**	**(32,64)**	**(10,13)**	**3**	**90**
PCSeg Tool-2	145	(22,43)	(7,9)	16	102
Saeedizadeh et al. [24]	161	(26,46)	(3,5)	16	192

*Some clusters were completely rejected out of the total of 45 clusters.

**Table 4:** Statistical results on a total of 364 (260+102) plasma cells.

Method	True Positive Rate or Recall (%)	Positive Predictive Value or Precision (%)	False Discovery Rate (%)	F_1_-Score (%)
PCSeg Tool-1	**81.66**	**76.56**	**23.44**	**79.03**
PCSeg Tool-2	54.72	65.88	34.12	59.78
Saeedizadeh et al. [24]	58.88	52.47	47.53	55.49

Further, we compared both the proposed methods, i.e., PCSeg Tool-1 (PCSeg Tool with *k*-means as nuclei mask for cluster cell segmentation) and PCSeg Tool-2 (PCSeg Tool with Ω_11_ phase of level set as nuclei mask for cluster cell segmentation) with existing cell segmentation methods relevant to our problem. These existing methods included *k*-means method, Chan-Vese active contour, Chan-Vese multiphase methods, and a recently described method by Saeedizadeh et al. [24]. *k*-means is a widely used method, while the proposed multiphase level set method in PCSeg Tool-1 and PCSeg Tool-2 is a refinement over standard active contour methods of Chan-Vese active contour [16] and Chan-Vese multiphase [29] methods. All these methods, i.e., *k*-means and standard active contour methods, have been used earlier on segmentation of cells other than plasma cells. The method of Saeedizadeh et al. [24] addresses specifically the plasma cell segmentation. Hence, all the above four methods were chosen for qualitative comparison with PCSeg Tool-1 and PCSeg Tool-2.

## Discussion

The *k*-means method provided many false positives, missed many PCs, and could not segment clusters of PCs (Fig 9). Similar was the case with standard active contour methods of Chan-Vese active contour and Chan-Vese multiphase methods. Since*k*-means, Chan-Vese active contour [16], and Chan-Vese multiphase [29] methods performed poorly on PC segmentation, quantitative results have been presented on only the rest of the three methods (Tables 3 and 4).

As compared to Saeedizadeh et al. [24], PCSeg Tool-1, and PCSeg Tool-2 performed far more superior to *k*-means and standard active contour methods (Tables 3 and 4). While PCSeg Tool-2 did not outperform [24] in the number correct PC cell segmentation, it did better with FDR compared to [24] which led to a very high number of 192 false positives with an FDR of 47.52% (Table 4). The method by [24] performed second best by segmenting 62% of single plasma cells and 64.5% of PC clusters (57.8% complete clusters and 6.7% partial clusters). However, one of the 4 plasma cells present in Fig 9 has been incorrectly segmented by [24]. On the other hand, PCSeg Tool-1 and PCSeg Tool-2 captured all 4 cells. This result shows that both the variants of PCSeg Tool (1 and 2) performed better in capturing the cells of interest.

This is an expected result because the methods specifically designed for plasma cell segmentation, i.e., PCSeg Tools 1 and 2 and [24] take into account the problems inherent to plasma cell segmentation. Further, these results establish that the method applicable on one cell type cannot be ported for segmentation of another cell type directly, i.e., no single method or segmentation pipeline can be applied to all the different cell types.

We also compared the performance of the above three segmentation tools with reference to Precision, Recall, and F1-score. While recall rate quantifies a tool’s performance with respect to false negatives (how many plasma cells were missed out in segmentation), precision rate informs us about the performance of the tool with respect to false positives, i.e., other cells segmented out as plasma cells. Thus, recall informs what ratio of correct plasma cells could be segmented, while precision tells how many of the cells segmented as plasma cells were erroneous. Thus, both precision and recall have a significance and both these are imbibed in the F_1_-score that should be as high as possible and informs about the overall performance of the segmentation tool. It is noted that recall of PCSeg Tool-1 is good as 81.66% compared to 54.72% of PCSeg Tool-2 and 58.88% of [24]. Precision of PCSeg Tool-1 is also good as 76.56% compared to 65.88% of PCSeg Tool-2 and 52.47% of [24]. Thus, as expected, PCSeg Tool-1 provides best F1-score of 79% compared to 59.78% of PCSeg Tool-2 and 55.49% of [24]. Thus, although PCSeg Tool-2 could not segment as many PCs as [24], it yielded more correct segmented cells compared to [24].

Overall, PCSeg Tool-1 performed best and provided better TPR, PPV, and smaller FDR. It was not only able to reduce the number of false negatives; it was also able to reduce false positives. For use in real clinical treatment, false negative rate of PCs should be low as well as false positives should be low because less than required number of chemotherapy sessions due to poor recall (or missing of large number of true PCs) or more than required number of chemotherapy sessions on aged people owing to high false discovery can prove to be fatal.

**Fig 10:**
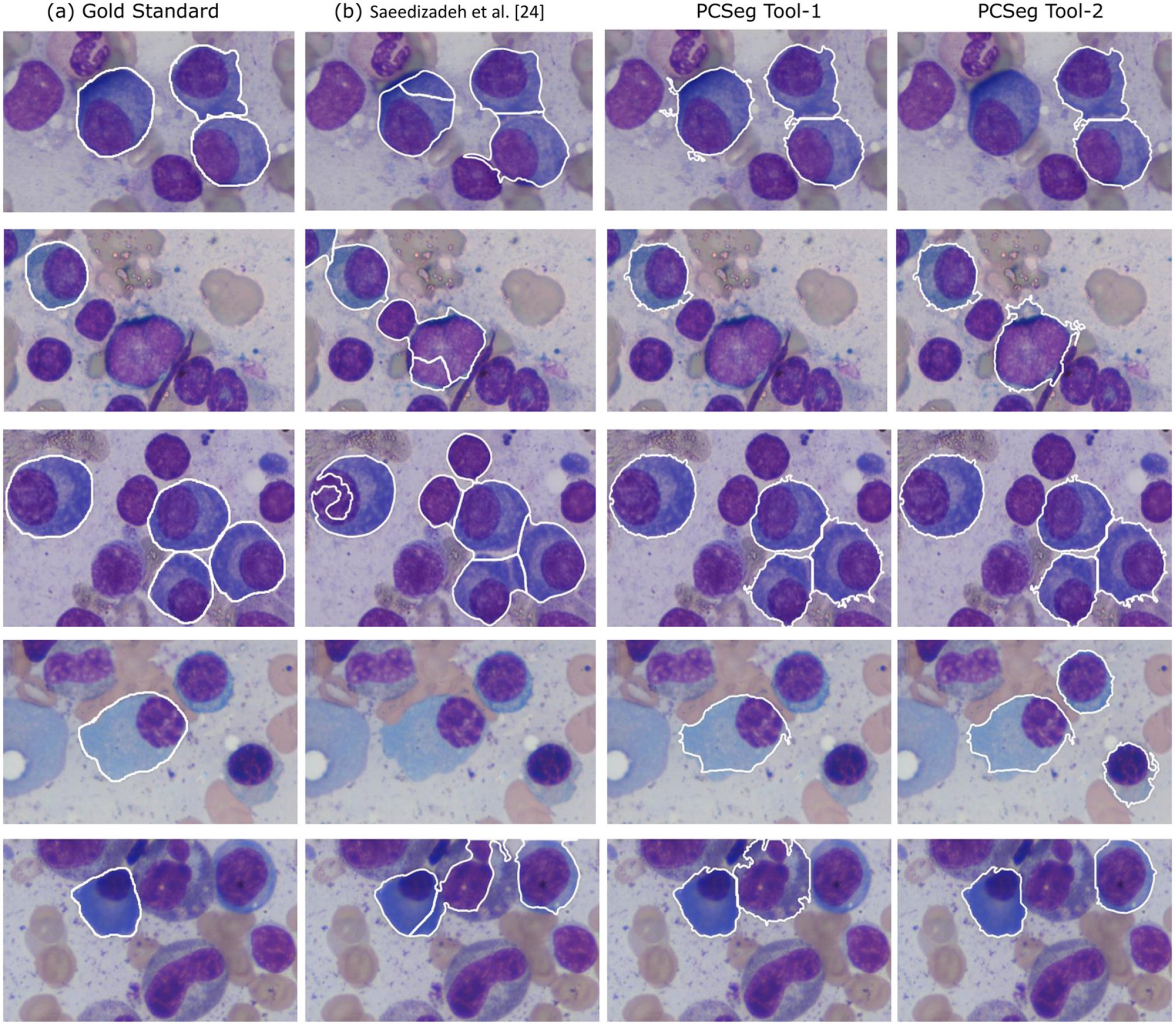
**Qualitative comparison of MM cell segmentation using different methods over five images:** (a) Gold standard (showing cells of interest with white outlines), (b) Saeedizadeh et al. [24] method, (c) PCSeg Tool-1, and (d) PCSeg Tool-2. White outlines in all figures (b)-(d) denote the outlines of regions segmented out. These regions are required to be compared with the regions contained within the white boundaries in Gold Standard shown in (a).

Fig 10 presents qualitative (visual) comparison of these methods on some more images. The good performance of PCSeg Tool-1 with *k*-means identified nuclei mask for cluster cell segmentation in the modified level set formulation implies that perhaps the proposed probability based and mean color vector based energy functionals in the multiphase level set, added with the robustness of the *k*-means on the level set output for nuclei mask for subsequent cluster cell segmentation, is playing a key role in the robust segmentation of PCs. Thus, we name the PCSeg Tool-1 as the final PCSeg Tool product for use with plasma cell segmentation.

## Conclusions and future work

In this paper, we designed, described, and implemented PCSeg tool for the segmentation of plasma cells from microscopic images. This tool has a robust pipeline consisting of modified multiphase level set method that utilizes statistical information about the probability densities of regions of interest (ROI) and the mean color vector of ROI in the color spaces in the multiphase level set. The level set stage removed the background and most of the unwanted cells. Only the stained single cells or the clusters of cells were retained after its application. Next, we tried two variations of PCSeg Tool: Tool-1 that utilized *k*-means based nuclei mask in the cluster cell segmentation and Tool-2 that utilized one of the phases of the level set for nuclei mask in cluster cell segmentation, where cluster segmentation was carried out with watershed and circular Hough transform and unwanted cells are completely removed in the post-processing stage of PCSeg Tool. PCSeg Tool-1 provided best results with better recall, precision, and F1-score. Further, the implemented PCSeg Tool-1 provided good results on segmentation of single isolated plasma cells as well as segmentation of plasma cells from cell clusters.

Recently, cell segmentation with deep learning (DL) has started picking up pace. However, as of now there are only a few papers with DL on cell segmentation [30–35]. Most of these methods have dealt with nucleus segmentation and so far, there is no paper on plasma cell segmentation using deep learning. Plasma cell segmentation is a more challenging problem compared to nucleus segmentation because (i) it requires both nucleus and cytoplasm segmentation, (ii) the color contrast of cytoplasm is sometimes very near to background, and (iii) cluster segmentation is also a problem because it can include a cluster of touching nuclei, touching nucleus with cytoplasm, touching cytoplasm of different cells, etc. Hence, recently proposed DL methods cannot be directly ported on this dataset. Solving the problem of plasma cell segmentation using DL is a challenging research problem that we plan to attempt in the near future. In addition, recent optimization/regularization based methods used in other domains of medical image segmentation similar to low rank and sparse decomposition method of [36] can also be explored in cell segmentation.
